# Comparative Analysis of the Spatiotemporal Evolution Patterns of Acoustic Emission Source Localization Under True Triaxial Loading and Loading-Unloading Conditions in Sandstone

**DOI:** 10.3390/s26010167

**Published:** 2025-12-26

**Authors:** Peng Chen, Shibo Yu, Hui Wang, Zhixiu Wang, Nan Li

**Affiliations:** 1 China-South Africa Joint Research Center for Development and Utilization on Mineral Resources, BGRIMM Technology Group, Beijing 100160, China; 2 School of Mines, China University of Mining and Technology, Xuzhou 221116, China; 3 State Key Laboratory for Fine Exploration and Intelligent Development of Coal Resources, China University of Mining and Technology, Xuzhou 221116, China

**Keywords:** true triaxial, loading-unloading, acoustic emission source localization, time-frequency conversion, waveform parameters

## Abstract

Microseismic/acoustic emission (AE) monitoring enables real-time, non-destructive observation of deformation and failure processes in rock during loading and unloading. Accordingly, this study designed two experimental schemes—sandstone loading and unloading—to comparatively investigate the spatiotemporal evolution characteristics of AE during sandstone failure under these distinct stress paths. Based on AE waveform time-frequency parameters and AE source location results obtained during testing, the failure evolution patterns of rock under both loading paths were analyzed. The results demonstrate that: (1) In both loading and load-unloading experiments, rock failure exhibited a distinct four-stage characteristic. Under pure loading conditions, failure concentrated near the point of catastrophic rupture, whereas unloading triggered premature rock fracturing, with a more pronounced AE response observed during the unloading phase. (2) For both loading paths, the dominant frequencies of AE waveforms were concentrated within the 0–200 kHz range. A distinct low-frequency (0–100 kHz), high-amplitude zone emerged prominently during Stage 4 in both cases. (3) AE source locations under load-unloading conditions revealed that during Stage 3—characterized by vertical loading combined with lateral unloading in the minimum principal stress direction—tensile failure cracks nucleated within the rock. Subsequently, during Stage 4 of the loading phase, these cracks propagated and coalesced, ultimately forming a macroscopic fracture surface on the sandstone specimen. (4) The AE source location results under pure loading failure conditions indicate that under uniaxial vertical loading, compression-shear failure fractures begin to develop within the rock mass during Stage 3. With continued loading in Stage 4, these shear fractures propagate through to the specimen surface, forming a through-going shear fracture plane.

## 1. Introduction

Regarding deformation and failure issues in rock during complex loading-unloading processes, scholars have investigated rock properties [1], stress states [2], and loading paths [3] to determine various rock strength parameters, failure modes, and stress–strain relationships. Field tests have further examined deformation characteristics, including rock mass displacement [4], stress concentration states [5], fracture development [6], and damage zones [7], yielding substantial research outcomes. Nevertheless, effective monitoring methods for rock deformation and failure under complex loading-unloading stress paths remain inadequate, hindering timely and accurate assessment of rock failure severity and effective early warning of potential rock failure-induced disasters.

Microseismic/Acoustic Emission (AE) monitoring is a geophysical technique that studies material deformation and fracture by detecting vibration waves emitted from rock rupture. Characterized by real-time, continuous, and non-destructive capabilities, AE monitoring has been widely applied in laboratory studies of rock failure signal responses in recent years [8,9,10,11,12,13,14,15,16,17,18,19,20]. Critically, AE source localization has evolved into a quantifiable diagnostic tool for internal damage evolution in rock: in laboratory settings, high-precision (<5 mm error) 3D positioning of micro-cracks enables direct correlation between fracture networks and stress–strain stages [21,22]; in engineering applications, real-time spatiotemporal mapping of rupture zones (e.g., in tunnel walls or coal pillars) provides actionable metrics for damage severity assessment [23,24]. In engineering practice, extensive research has explored microseismic/AE time-frequency response patterns under influences such as geological structures [11], excavation disturbances [12], and stress concentrations [13]. Investigations into spatiotemporal evolution laws of microseismic activities associated with rock bursts and rockbursts [14,15,16]—including amplitude-frequency characteristics, frequency bands, and energy distributions [17,18,19]—have provided theoretical foundations for predicting dynamic rock disasters. Numerous monitoring and early-warning indicators/methods have been proposed [20,25,26], such as microseismic energy-frequency correlations [25] and seismic moment [26]. However, despite the validated effectiveness of AE technology in rock failure monitoring, comparative studies under complex loading-unloading stress paths remain insufficient, and an integrated analytical framework linking “time-frequency parameters—location accuracy—damage evolution” is lacking.

To address these gaps, this study focuses on the true-triaxial (polyaxial) stress environment, which better replicates in situ rock stress conditions, to investigate spatiotemporal failure evolution during rock loading and load-unloading processes. We designed true-triaxial AE monitoring experiments on sandstone specimens under both loading and load-unloading conditions, systematically comparing—for the first time—spatiotemporal AE distribution differences between these two representative stress paths, thereby overcoming limitations of previous single-path studies. The Akaike Information Criterion (AIC) algorithm was employed to optimize waveform arrival-time picking accuracy, combined with the Simplex location algorithm to achieve high-precision AE source localization—resolving significant positioning errors under complex stress paths. A comprehensive analytical framework was established, integrating “time-frequency parameter evolution—full-process stage division—spatial fracture characteristics.” By correlating source counts with localization results, this study reveals the differential influences of loading versus load-unloading stress paths on sandstone failure modes, providing more robust theoretical underpinnings for precise early warning of dynamic rock disasters.

## 2. True Triaxial Unloading AE Monitoring Experiment

### 2.1. Experimental Equipment

**Figure 1:**
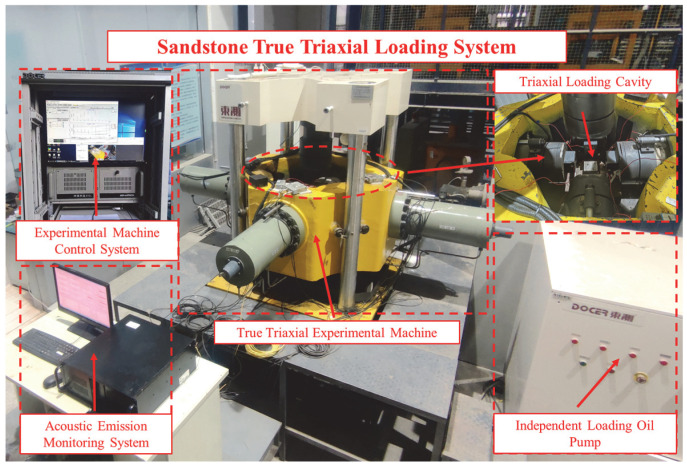
Sandstone true triaxial loading system.

The sandstone true-triaxial loading system is a proprietary true-triaxial testing machine independently developed by China University of Mining and Technology. As illustrated in Figure 1, this testing machine comprises a main loading frame, vertical and horizontal loading modules, independent hydraulic power units, a triaxial loading chamber, AE sensor mounting platens, and computer control software. The system employs electro-hydraulic servo control, with maximum loading capacities of 1600 kN in the vertical direction and 600 kN in the horizontal direction. Loading modules in different orientations operate independently to enable concurrent loading/unloading without mechanical interference.

The machine is equipped with interchangeable loading platens accommodating cubic specimens of three standard dimensions: 200 mm, 150 mm, and 100 mm. Through configurable programming, it executes diverse experimental protocols—including monotonic loading, stepwise loading, and cyclic loading-unloading—under uniaxial, biaxial, and true-triaxial stress conditions. Dedicated platens with integrated mounting fixtures ensure direct coupling between acoustic emission (AE)/ultrasonic sensors and sandstone specimens during testing. Each platen supports four sensors, enabling synchronous monitoring with up to 24 sensors in experimental configurations. To minimize interfacial friction between specimens and platens, grease-lubricated polyvinyl chloride (PVC) film is applied at contact interfaces during testing.

The acoustic emission (AE) monitoring system employed a 24-channel Micro-II Digital AE acquisition host (Physical Acoustics Corporation, MISTRAS Group, Princeton, NJ, USA), preamplifiers, Nano30 AE sensors, and ROCKTEST for Express-8 software (E5.90) for data acquisition and analysis. This integrated system enables real-time capture of AE time-domain parameters and raw waveforms, spectral analysis of AE waveforms, and event source localization. Prior to testing, sensor coupling quality and system accuracy were validated through pencil lead break tests.

**Figure 2:**
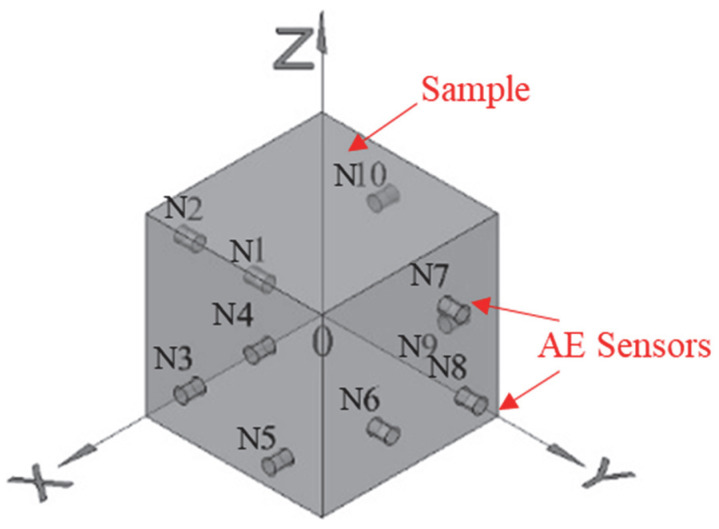
Spatial layout of AE sensors.

During experiments, the preamplifier gain was set to 40 dB, with a detection threshold of 40 dB, sampling rate of 1 MHz, Pre-trigger Hold-off Time (PHT) of 200 μs, Hit Definition Time (HDT) of 400 μs, and Hit Lockout Time (HLT) of 500 μs. Ten Nano30 AE sensors were deployed in this study, featuring a frequency response range of 150–400 kHz—adequate for monitoring AE activity in sandstone specimens. Ultrasonic couplant was applied to sensor contact surfaces to enhance coupling efficiency between sensors and specimens. Figure 2 illustrates the spatial arrangement of sensors for AE localization monitoring during sandstone loading and load-unloading failure tests. Sensor coordinates and AE acquisition parameters are detailed in Table 1.

### 2.2. Sample Preparation

**Figure 3:**
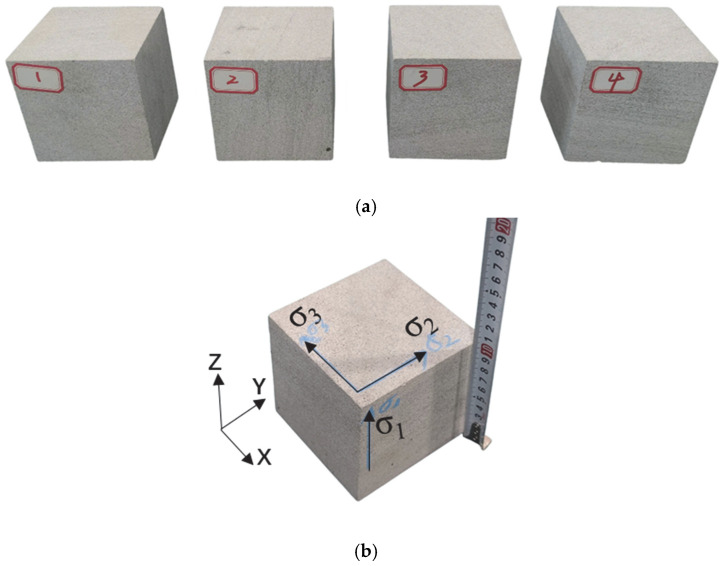
Sample for true triaxial loading and loading-unloading of sandstone: (**a**) pre-prepared original sample; (**b**) Experimental sample.

The experimental rock specimens were sandstone blocks exhibiting high density and homogeneity. As shown in Figure 3, four cubic specimens of dimensions 100 mm × 100 mm × 100 mm were precision-machined from a large sandstone block. Specimens were processed strictly in accordance with the recommended standards of the International Society for Rock Mechanics (ISRM), maintaining dimensional tolerances within ±0.2 mm and surface flatness deviations below 0.05 mm (Table 2). The finished sandstone specimens displayed uniform fine-grained texture, smooth surfaces, and no visible natural joints or fractures.

Density and ultrasonic wave velocity measurements were conducted on all candidate specimens, with those exhibiting minimal property variability selected for true-triaxial loading/unloading AE localization monitoring experiments. In the experimental configuration, the principal stress directions were defined as follows: σ_1_ aligned with the Z-axis, σ_2_ with the Y-axis, and σ_3_ with the X-axis.

Prior to conducting true-triaxial load-unloading failure AE monitoring tests on sandstone, its mechanical parameters were predetermined. Initially, sandstone specimens were sampled and tested to determine key mechanical properties, including uniaxial compressive strength, tensile strength, elastic modulus, Poisson’s ratio, cohesion, and internal friction angle.

### 2.3. Experimental Scheme

The core mechanical effect of roadway excavation is “radial unloading coupled with vertical stress redistribution,” where surrounding rock transitions from a triaxial isotropic stress state to an anisotropic state. During excavation, radial stress release and vertical stress concentration initiate simultaneously upon formation of the excavation face. This stress path is precisely replicated through coordinated axial-confining pressure control in true-triaxial testing, enabling accurate simulation of this “unloading-loading coupling” process. This approach overcomes the limitation of conventional single-path loading or unloading tests, which fail to capture the actual in situ stress evolution. The simulated stress path employs a three-stage protocol—“pre-loading equilibrium → initial stress stabilization → σ_1_ loading + σ_3_ unloading + constant σ_2_”—to model the dynamic stress evolution in roadway surrounding rock during excavation.

**Figure 4:**
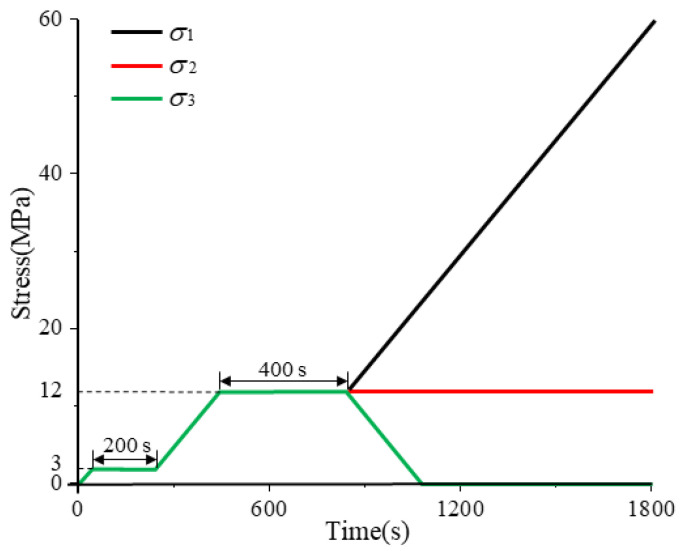
Experimental scheme for loading-unloading failure of sandstone.

In engineering practice, roadway excavation disrupts the in situ stress field, transforming the surrounding rock from a triaxial isotropic state to an anisotropic state characterized by vertical loading, radial unloading, and unchanged strike-direction stress. To replicate this, a rock load-unloading stress path was designed by configuring differential axial–confining pressure relationships in true-triaxial testing (Figure 4). The experimental procedure comprised as follows: Pre-loading: Triaxial compression at 0.05 MPa/s to 3 MPa, held constant for 200 s; Formal loading: Constant-rate loading (0.05 MPa/s) to simultaneously establish σ_1_ = σ_2_ = σ_3_ = 12 MPa, held for 400 s; σ_1_ further loaded at 0.05 MPa/s until specimen failure; σ_2_ maintained constant; σ_3_ unloaded at 0.05 MPa/s simultaneously with σ_1_ loading] to 0 MPa. Acoustic emission (AE) localization monitoring was continuously performed throughout the sandstone specimen’s deformation and failure process under this load-unloading protocol.

During preloading, the specimen is uniformly loaded triaxially to 3 MPa at a constant rate, followed by a 200 s constant load period. This step simulates the initial equilibrium state of the in situ stress field, ensuring uniform stress distribution inside the specimen, eliminating initial pore and stress hysteresis effects, and restoring the stress foundation of the surrounding rock prior to excavation. For formal loading, the specimen is continuously loaded triaxially to 12 MPa and maintained under constant load for 400 s, which simulates the triaxial isostatic stress environment of the surrounding rock before tunnel excavation (i.e., the in situ stress state) and ensures the specimen reaches an initial stable stress level consistent with engineering practice. In the subsequent loading phase, σ_1_ is further increased, σ_3_ is unloaded to 0 MPa, and σ_2_ is kept constant. This stage corely simulates the excavation unloading effect: after tunnel excavation, a free surface is formed in the radial direction (corresponding to σ_3_), leading to the release of in situ stress (hence σ_3_ is unloaded to 0 MPa); in the vertical direction (corresponding to σ_1_), stress cannot be released due to the self-weight of the overlying strata and lateral constraints, and instead redistributes with the deformation of the surrounding rock (hence σ_1_ is further loaded); in the strike direction (corresponding to σ_2_), stress release is limited because the tunnel length is much larger than its cross-sectional dimensions, maintaining the initial stable state (hence σ_2_ is kept constant). Eventually, a triaxial anisostatic stress field characterized by “vertical loading, radial unloading, and strike constant load” is established.

After tunnel excavation, the surrounding rock does not remain stable permanently. Adjacent mining and excavation activities will disrupt the balanced stress field, leading to re-increased vertical stress in local areas (e.g., 10–50 m behind the excavation face) and forming secondary stress concentration. This stress path fixes σ_2_ and σ_3_ to simulate the stabilized radial-strike stress field, while only increasing σ_1_ to simulate secondary loading, which accurately reproduces the mechanical process of “stable surrounding rock → local stress concentration → secondary failure”. The stress configuration (σ_3_ = 6 MPa, σ_2_ = 12 MPa, initial σ_1_ = 24 MPa) is consistent with the actual stress ratio of underground tunnels at a burial depth of 500–1000 m. Through three stages—“preloading equilibrium → triaxial stepwise loading stabilization → σ_1_ secondary loading with σ_2_/σ_3_ constant load”—this stress path simulates the secondary stress concentration of surrounding rock caused by mining disturbance after the tunnel excavation stabilizes.

**Figure 5:**
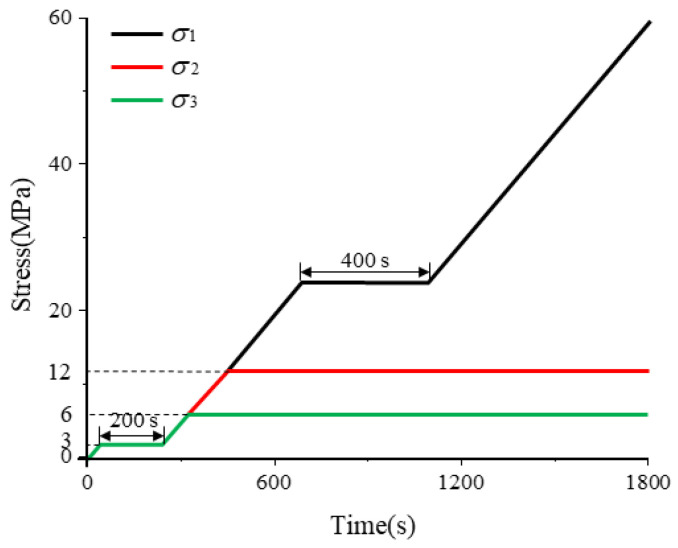
Experimental scheme for the loading failure of sandstone.

For comparison, after tunnel excavation, the surrounding rock behind the excavation face is reloaded vertically due to adjacent mining activities, resulting in local stress concentration and further damage to the stabilized surrounding rock. Therefore, a triaxial stress state of “vertical loading, radial constant load, and strike constant load” is designed for the rock specimen. Corresponding relationships between different axial and confining pressures in the true triaxial test are set as shown in Figure 5. Firstly, the sandstone specimen is preloaded: triaxially loaded uniformly to 3 MPa at a rate of 0.05 MPa·s^−1^, followed by a 200 s constant load period. For formal loading: the specimen is loaded uniformly at 0.05 MPa·s^−1^—σ_3_ (minimum principal stress) is loaded to 6 MPa and held constant, σ_2_ (intermediate principal stress) is loaded to 12 MPa and held constant, and σ_1_ (maximum principal stress) is loaded to 24 MPa and maintained for 400 s. Subsequently, σ_1_ is continuously loaded at 0.05 MPa·s^−1^ until the specimen undergoes complete failure. Acoustic emission (AE) location monitoring is performed throughout the entire loading-unloading deformation and failure experiment of the sandstone specimen.

During preloading, the specimen is uniformly loaded triaxially to 3 MPa and held at a constant load for 200 s, which simulates the initial equilibrium of in situ stress and eliminates the influence of initial defects in the specimen. For formal stepwise loading, σ_3_ = 6 MPa, σ_2_ = 12 MPa, and σ_1_ = 24 MPa are applied, followed by a 400 s constant load period. This stage simulates the stress stabilization state of the surrounding rock after tunnel excavation through initial deformation adjustment: radial stress σ_3_ forms a residual stress of 6 MPa due to tunnel support or self-stabilization of surrounding rock, strike stress σ_2_ maintains the in situ stress level of 12 MPa, and vertical stress σ_1_ reaches an initial stable stress of 24 MPa. In the subsequent loading phase, σ_1_ is further increased while σ_2_ and σ_3_ remain constant. This simulates the local stress concentration caused by superimposed vertical stress in the surrounding rock behind the excavation face, which is affected by adjacent mining activities such as tunnel extension and stopping operations. In contrast, radial and strike stresses remain stable due to the completion of initial adjustment, ultimately leading to further failure of the surrounding rock under the condition of “vertical secondary loading and radial-strike constant load”.

Acoustic emission (AE) location monitoring is performed throughout the entire loading-unloading deformation and failure experiments of sandstone specimens under the two stress paths. By analyzing the spatiotemporal distribution characteristics of AE signals, the dynamic processes of crack initiation, propagation, and coalescence inside the specimens are captured, providing data support for revealing the failure mechanism of the surrounding rock under different engineering scenarios.

The stepwise loading-constant load design takes into account the phased characteristics of sandstone failure, including compaction of primary fractures, elastic deformation, microcrack propagation, and macroscopic instability. Through the stepwise design of preloading at 3 MPa and formal loading at 12/24 MPa, fracture behaviors under different stress levels can be isolated to avoid signal superposition. The constant load phase provides a sufficient time window for mechanical responses under each stress level, ensuring that AE signals can fully characterize the fracture characteristics of the corresponding stage.

The differentiated constant load durations are attributed to the positive correlation between stress level and AE quiet period. The AE quiet period refers to the duration during which crack activity of rock mass is mild under specific stress, and the signal count rate decreases to the background level, whose length increases with the rise in stress. Under low-stress preloading (3 MPa), the main process is the closure of primary fractures, with rapid signal attenuation; 200 s can cover the quiet period, ensuring clear signal demarcation. Under medium-high stress formal loading (12/24 MPa), the specimen enters the elastoplastic transition zone, where crack propagation and slip persist, resulting in a long signal stabilization period; 400 s can ensure the end of the quiet period and avoid interference with subsequent experiments.

Meanwhile, this design meets the requirements of stress homogenization and creep stabilization. Rock exhibits time-dependent rheological effects (creep and relaxation). Under low-stress preloading (3 MPa), sandstone is in the limited creep stage of primary fracture compaction; a 200 s constant load can achieve elastic recovery and initial creep stabilization, eliminating transient loading disturbances. Under medium-high stress formal loading (12/24 MPa), the stress is close to the proportional limit of sandstone, and the specimen enters the logarithmic creep stage characterized by crack slip and secondary fracture initiation; a 400 s constant load is required to ensure uniform stress distribution and stable creep deformation.

## 3. Analysis of Experimental Results

### 3.1. Time Domain Response Results of AE

The core basis for selecting a single specimen for preliminary analysis lies in the significant similarity of mechanical responses and failure characteristics among multiple sandstone specimens under the same loading-unloading path. The signal variation trends of 10 acoustic emission (AE) channels also maintain high consistency, with only minor numerical differences and no systematic deviations. Based on this experimental phenomenon, selecting a typical specimen and channels for preliminary analysis can effectively eliminate the interference of random errors, focus on revealing the core correlation laws between rock mechanical behaviors and AE signals during the loading-unloading process, and the conclusions are of certain representativeness and reliability.

However, it should be clarified that the analysis at this stage focuses on the qualitative revelation of laws rather than statistical quantitative verification. The limited number of specimens and the lack of variability analysis may result in the inability of the results to fully reflect the global lithological differences in sandstone (e.g., the natural heterogeneity of microfracture development degree) and make it difficult to quantify the statistical confidence level of the experimental results. Future research will be improved in the following aspects: (1) Increase in the number of specimens and design multiple sets of parallel experiments to systematically analyze the variability of mechanical parameters among different specimens; (2) Adopt statistical methods to quantify the degree of data dispersion and clarify the reliability range of experimental results; (3) Combine the natural structural properties of sandstone, correlate the intrinsic relationship between lithological heterogeneity and experimental variability, and enhance the universality and engineering application value of the research conclusions.

**Figure 6:**
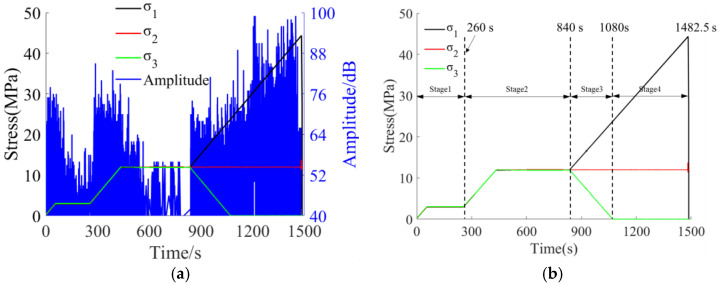
Relationship between stress and AE response under loading-unloading condition in sandstone: (**a**) AE response; (**b**) Stage division.

In the sandstone loading-unloading failure experiment, the entire process is divided into four stages according to the stress loading path. The first two stages each include a loading process and a constant load process; the third stage is a loading-unloading stage, and the fourth stage is a continuous loading process until the specimen fails. The variation trends of sandstone loading stress and AE amplitude (maximum amplitude value) with time during the experiment are shown in Figure 6a, and the stage division of the entire loading-unloading experiment process is shown in Figure 6b.

Stage 1: Due to the simultaneous triaxial loading, the fine pores and fractures inside the sandstone are rapidly compacted and closed. The internal stress adjustment of the specimen is relatively intense, and the deformation is obvious, resulting in relatively active AE signals. During the constant load phase, the triaxial stress inside the specimen no longer continues to increase; the internal stress adjustment of the sandstone gradually slows down, the AE signals tend to be calm, the number of signals gradually decreases, and the amplitude gradually decreases. The maximum amplitude of sandstone in this stage is 77 dB, indicating that no obvious damage occurs to the sandstone mass at this stage.

Stage 2: During the formal loading of sandstone, the fine pores and fractures inside the sandstone specimen are continuously compacted under external forces, and the specimen begins to transition from the compaction stage to the elastic stage. The AE signals also change from a state of high quantity and amplitude to a state of low quantity and amplitude. Subsequently, during the constant load phase, the triaxial stress reaches a triaxial isostatic state, the internal stress of the sandstone specimen no longer increases, the number of AE signals gradually decreases, and the amplitude gradually decreases. Due to the stable stress during the constant load process, the number of generated AE signals is the least, and the amplitude is relatively low. The maximum amplitude of sandstone in this stage is 85 dB, indicating that a small amount of fracture begins to occur in the sandstone mass, and the existing fractures start to develop.

Stage 3 (Loading-Unloading Stage): Due to the lateral constant load and vertical loading, the vertical microfractures inside the sandstone specimen are continuously compacted and closed. However, the stress in the σ_3_ direction undergoes tensile failure due to unloading, forming new fine fractures, and the existing fractures begin to develop. This leads to an increase in the number of AE signals and an increase in amplitude, with the maximum amplitude significantly higher than that of the previous stage. After the completion of unloading, the AE amplitude begins to decrease. The maximum amplitude of sandstone in this stage reaches 82 dB, indicating that a large number of new fine fractures have been generated inside the sandstone, the existing fractures have started to develop, propagate, and coalesce, and the integrity of the sandstone specimen has been greatly reduced.

Stage 4: Loading until the sandstone specimen undergoes unstable failure. In this stage, the number of generated AE signals is large, and the amplitude is high, with the maximum amplitude close to that of the previous stage. The maximum amplitude of sandstone in this stage is as high as 99 dB. At this time, the elastic energy accumulated inside the sandstone is rapidly released, resulting in a large amount of deformation, and the sandstone specimen fails unstably.

**Figure 7:**
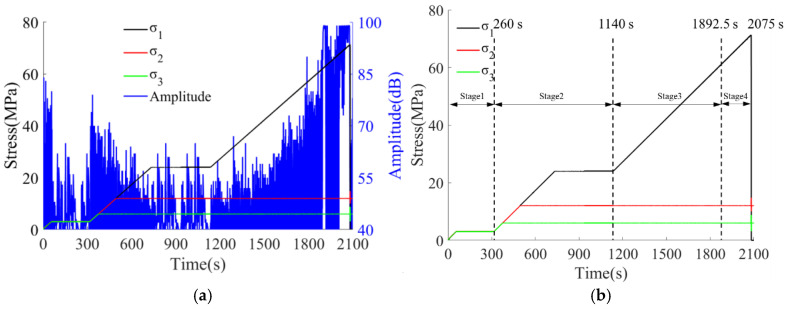
Relationship between stress and AE response during loading of sandstone: (**a**) AE response; (**b**) stage division.

Similarly, in the sandstone loading failure experiment, the entire process is divided into four stages based on the stress loading path and acoustic emission (AE) response results. The first two stages each consist of a loading phase and a constant load phase, while the third and fourth stages involve continuous loading until the specimen fails. The variation trends of sandstone loading stress and AE amplitude (maximum amplitude value) with time during the experiment are illustrated in Figure 7a, and the stage division is presented in Figure 7b.

Stage 1: During preloading, the sandstone is subjected to simultaneous triaxial loading, leading to the compaction and closure of inherent primary fine pores and fractures. Significant stress adjustment and deformation occur within the specimen, resulting in active AE signals characterized by high quantity and amplitude. In the constant load phase, triaxial loading ceases, and the internal stress adjustment of the sandstone slows down. The AE signals tend to stabilize, with a gradual decrease in both signal count and amplitude. The maximum amplitude of sandstone in this stage is 83 dB, indicating no obvious fracture generation in the sandstone mass.

Stage 2: During formal loading, the remaining fine pores and fractures inside the sandstone are further compacted and closed under external forces. Initially, AE signals are abundant with high amplitude; as the specimen enters the elastic stage, the signal count decreases, and the amplitude reduces. In the constant load phase, the triaxial stress reaches a stable state. Due to stress stabilization, the number of generated AE signals is minimized with relatively low amplitude. The maximum amplitude of sandstone in this stage is 80 dB, suggesting that only a negligible number of fine fractures are formed in the sandstone mass.

Stage 3: During this stage, AE signals generated during specimen loading are highly active, with a continuous increase in both signal count and amplitude values generally higher than those in the previous stage. As the axial stress continues to rise, the sandstone specimen undergoes further compressive deformation, and a large number of new fine fractures are initiated inside the rock.

Stage 4: Existing fractures begin to initiate, propagate, and coalesce, leading to a rapid increase in both the count and amplitude of AE signals. At 2075.0 s, the sandstone specimen reaches a peak strength of 71.10 MPa, followed by unstable failure.

Based on the comprehensive analysis above, the waveform amplitude response laws in the first two stages are basically consistent throughout both the loading-unloading experiment and the loading experiment. However, there are significant differences between the third and fourth stages. In the third stage, the acoustic emission (AE) response level in the unloading phase of the loading-unloading experiment is significantly higher than that in the loading experiment. In contrast, in the fourth stage, the AE amplitude in the loading experiment is notably higher than that in the unloading phase of the loading-unloading experiment. This indicates that the fracture behaviors inside the sandstone differ in these two stages.

### 3.2. Time-Frequency Analysis of Typical Waveforms in the Loading-Unloading Process Based on Hilbert–Huang Transform (HHT)

**Figure 8:**
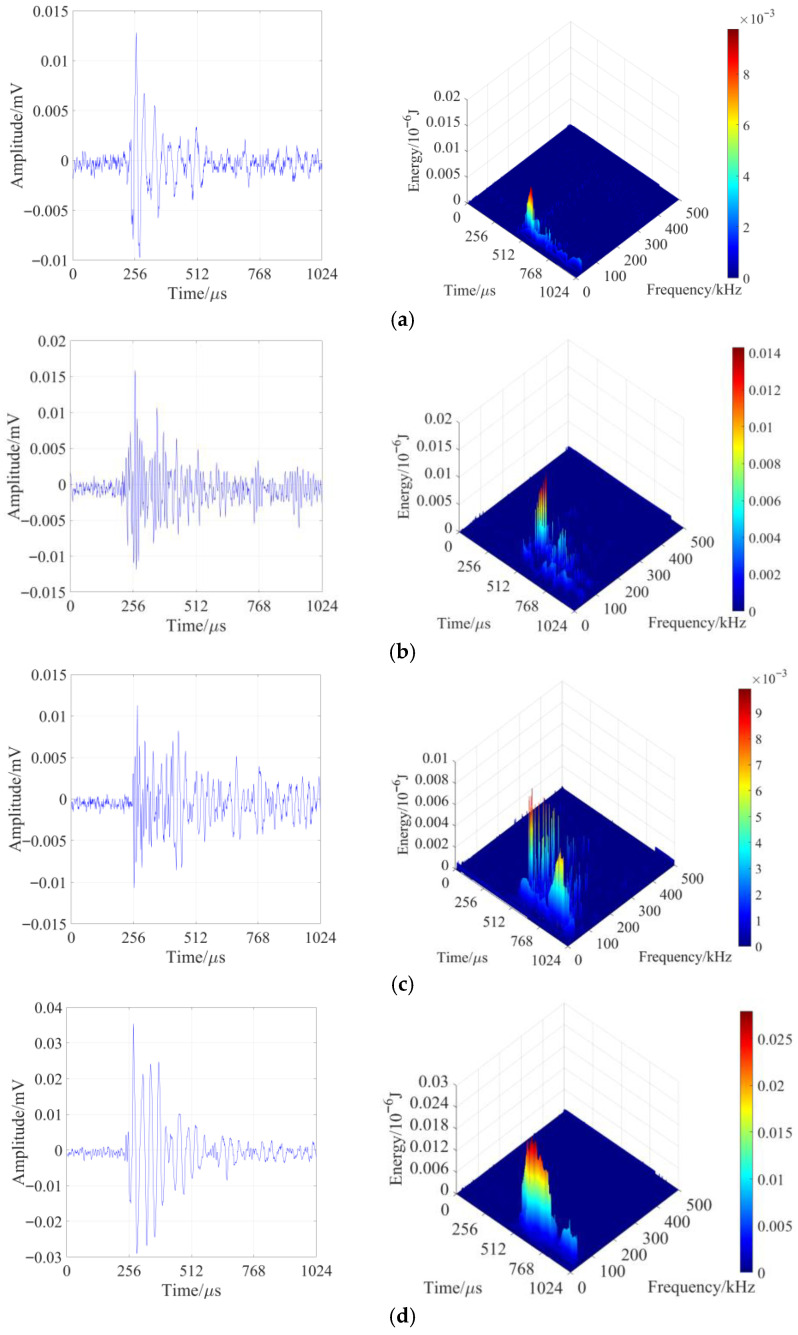
Typical waveform time-frequency conversion results for each stage of the loading-unloading process: (**a**) Typical waveform in stage 1; (**b**) Typical waveform in stage 2; (**c**) Typical waveform in stage 3; (**d**) Typical waveform in stage 4.

To analyze the frequency-domain characteristics of the waveforms, a single representative waveform was selected from each of the four stages of the unloading experiment for Hilbert–Huang Transform (HHT) analysis [27]. Given the non-stationary and nonlinear nature of stress-wave signals during rock unloading, HHT—particularly with CEEMDAN [28,29] (Complete Ensemble EMD with Adaptive Noise) as the decomposition step—was preferred over conventional Fourier- or wavelet-based methods [30,31]. Unlike Fourier analysis, which assumes global stationarity, or wavelet transforms, which depend on the choice of basis functions, HHT provides an adaptive, data-driven decomposition into intrinsic mode functions (IMFs), enabling high-resolution time–frequency representation via the Hilbert spectrum. CEEMDAN further mitigates mode mixing and improves mode separation compared to standard EMD. Nevertheless, HHT remains sensitive to noise and endpoint effects; to address this, signal extension (e.g., symmetric mirroring) and careful IMF selection criteria (e.g., eliminating spurious IMFs via energy thresholding and orthogonality checks) were applied. As shown in Figure 8, the marginal Hilbert spectrum for each stage reveals a clear peak in energy distribution; the corresponding frequency is identified as the dominant frequency of the waveform.

As shown in Figure 8, the frequency corresponding to the maximum energy is extracted and regarded as the dominant frequency of the waveform. For Waveform 1, the dominant frequency is distributed at 30 kHz, and the frequency band is concentrated within 0–100 kHz. For Waveform 2, the dominant frequency is at 90 kHz, while the frequency band is relatively scattered, with some low-frequency and high-energy information still present in the 0–100 kHz range. For Waveform 3, the dominant frequency is 86 kHz, the frequency band becomes more scattered, and an obvious secondary dominant frequency phenomenon is observed, with the secondary dominant frequency being 41 kHz. The dominant frequency extracted in Stage 4 is distributed at 25 kHz. It is evident that with the progression of the experiment, the frequency distribution of the waveforms shows a trend of “concentration → scattering → concentration”.

### 3.3. Results of Time-Frequency-Amplitude Response Throughout the Entire Experimental Process

**Figure 9:**
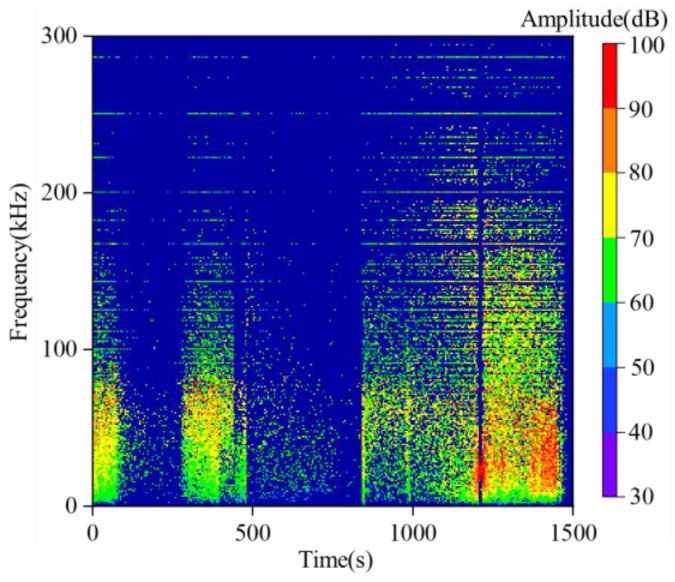
Time-frequency-amplitude evolution of source waveform under unloading condition in sandstone.

**Figure 10:**
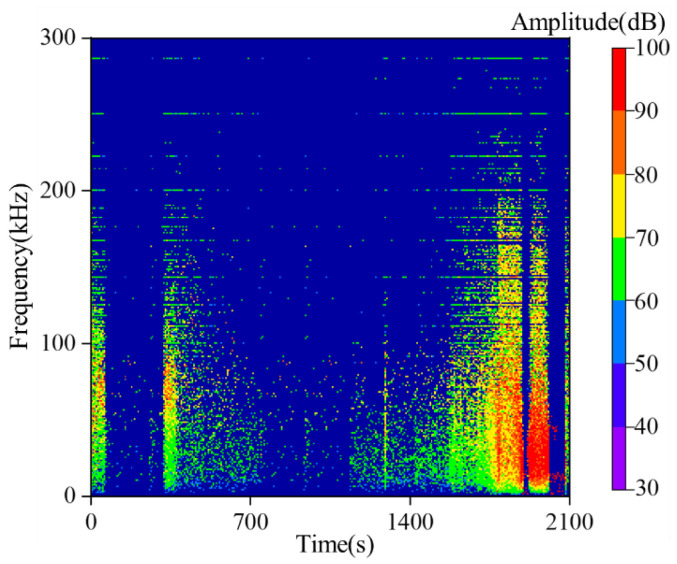
Time-frequency-amplitude evolution of source waveform under loading conditions in sandstone.

To further characterize the temporal sequence features of frequency information throughout the entire experimental process, we implemented programming in MATLAB 2019 to perform batch extraction of the dominant frequencies of all acoustic emission (AE) waveforms during the experiment via the Hilbert–Huang Transform (HHT), while simultaneously recording the waveform time and maximum waveform amplitude. The statistical results of the AE time-frequency-amplitude responses for the loading-unloading experiment and the loading experiment are presented in Figure 9 and Figure 10, respectively.

Throughout the entire loading-unloading experiment, the frequencies of acoustic emission (AE) signals generated by sandstone fracture are mainly distributed in the range of 0–300 kHz, with 90% of the waveform signals falling within 0–200 kHz. In terms of amplitude, it is observed that the amplitudes of source waveforms are primarily distributed between 0 and 100 dB. High-amplitude source waveform signals are concentrated in the 0–100 kHz frequency band, and as the stress continues to increase, the frequency of high-amplitude waveforms exhibits a decreasing trend. During the sandstone loading-unloading process, two low-frequency and high-amplitude regions are formed with the progression of sandstone fracture, which indicates that both unloading and loading can indeed cause severe damage to the sandstone.

Time-frequency-amplitude analysis via the Hilbert–Huang Transform (HHT) was performed on the source waves generated by sandstone failure during the true triaxial loading process. It was found that the frequencies of acoustic emission (AE) signals induced by sandstone failure under loading are also distributed in the range of 0–300 kHz. In terms of amplitude, high-amplitude source waveform signals are concentrated within the 0–100 kHz frequency band, and as the stress continues to increase, the frequency of high-amplitude waveforms also exhibits a decreasing trend. The difference is that during the sandstone loading process, only one low-frequency and high-amplitude region is formed with the progression of sandstone fracture. This indicates that continuous loading only causes one severe damage to the sandstone, leading to its unstable failure.

### 3.4. Analysis of RA-AF Values Throughout the Entire Experimental Process

**Figure 11:**
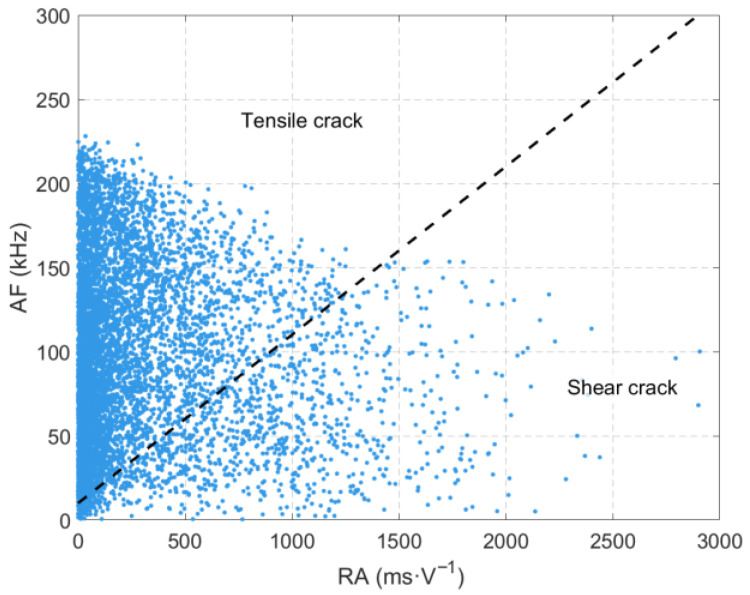
RA-AF value of the source waveform during sandstone loading-unloading.

The generation of low-frequency and high-amplitude signals mentioned in Section 3.3 is closely related to the formation and propagation of macroscopic cracks inside the rock. As shown in Figure 11, when the rock undergoes tensile failure, rapid crack propagation leads to the sudden separation of a relatively large volume of rock, releasing a substantial amount of elastic energy and generating acoustic emission (AE) signals with concentrated energy and low dominant frequency. Under unloading conditions, particularly during the unloading process in the direction of the minimum principal stress (σ_3_), significant tensile stress is induced, promoting the formation of a large number of tensile cracks inside the specimen.

**Figure 12:**
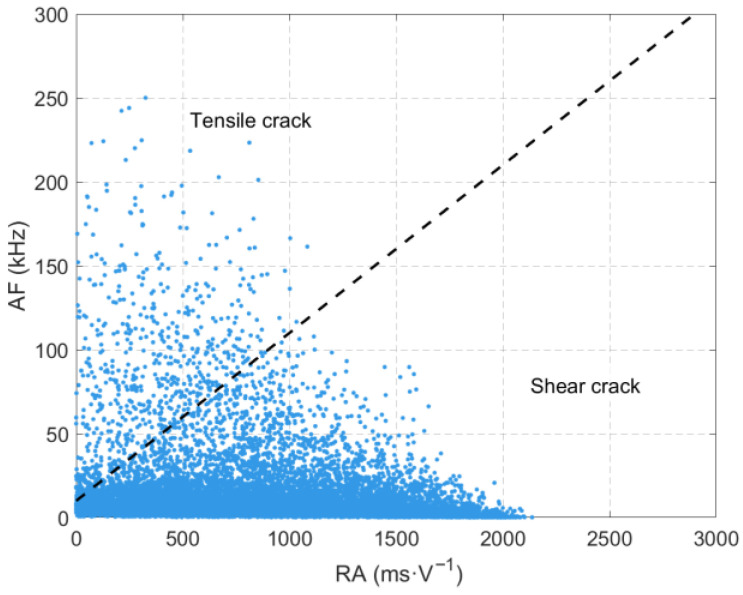
RA-AF value of the source waveform during sandstone loading.

During the sandstone loading process, only one low-frequency and high-amplitude region is formed as the sandstone fractures. As shown in Figure 12, unlike the unloading condition, the failure under pure loading is mainly dominated by the shear mechanism. Under high confining pressure, shear cracks are primarily formed inside the rock, generating relatively high-frequency acoustic emission (AE) signals. When the stress approaches the peak strength, a large number of shear cracks interconnect and form a macroscopic failure surface, where rapid energy release induces the generation of low-frequency and high-amplitude signals. This single low-frequency and high-amplitude region reflects the process of damage accumulation inside the sandstone under continuous loading until eventual sudden unstable failure, which is mainly manifested as a compression-shear failure mechanism. In contrast, under unloading conditions, the presence of tensile stress promotes earlier damage development and a more complex failure mode. This indicates that continuous loading results in a single severe damage event, leading to the unstable failure of the sandstone. The study found that the time-frequency-amplitude characteristics of source waveforms are closely related to sandstone failure; thus, the variation laws of the time-frequency-amplitude of sandstone failure source waveforms can be used as precursor information for sandstone failure.

## 4. Characteristics of the Spatiotemporal Evolution of AE Source Localization

The acoustic emission (AE) location data processing workflow in this study referenced the method described in Li [29], consisting of four key steps: (1) AE event identification; (2) valid event screening; (3) AE event waveform arrival time picking based on the Akaike Information Criterion (AIC) algorithm; (4) AE location event inversion based on the simplex method. This section analyzes the spatiotemporal characteristics of AE events throughout the entire process of the loading and loading-unloading physical experiments.

### 4.1. Localization Accuracy Calibration

Accurate source localization is a prerequisite for meaningful interpretation of the spatiotemporal evolution of AE activity. Potential sources of error—including uncertainties in the initial P-wave velocity model and variations in channel sensitivity—were systematically addressed. Given the relatively small scale and homogeneous lithology of the sandstone specimens (100 mm × 100 mm × 100 mm), an isotropic, homogeneous velocity model was adopted. The P-wave velocity (≈3850 m/s) was determined from first-arrival measurements across multiple sensor pairs and validated via ultrasonic pulse transmission tests.

To quantify the practical localization accuracy of the system, in situ lead-break (pencil-lead fracture) calibration tests were conducted after the main experiment, following the ASTM E1106-17 standard [32]. Nine lead breaks were performed at three predefined locations (near the top (75 mm, 75 mm, 100 mm), mid-height (0 mm, 25 mm, 50 mm), and bottom of the specimen (75 mm, 100 mm, 20 mm)), with three repetitions at each position to assess repeatability.

**Figure 13:**
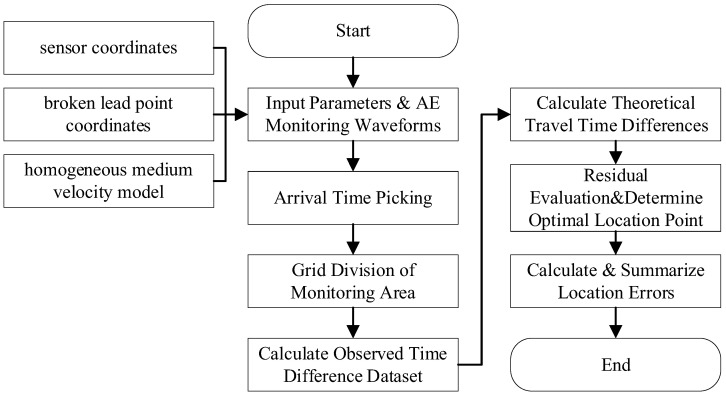
Flow chart of lead fracture location and error calculation based on the grid search method.

Notably, for this calibration phase, the grid-search method (see Figure 13 for workflow details) was employed for source location. This choice was motivated by its robustness in avoiding local minima—particularly advantageous for low-noise, high-SNR calibration signals where global optimality is preferred over computational speed. Additionally, grid search is computationally intensive and thus unsuitable for large-scale event processing (e.g., thousands of AE events) its use here is entirely appropriate given the limited number of calibration events (*n* = 9).

**Figure 14:**
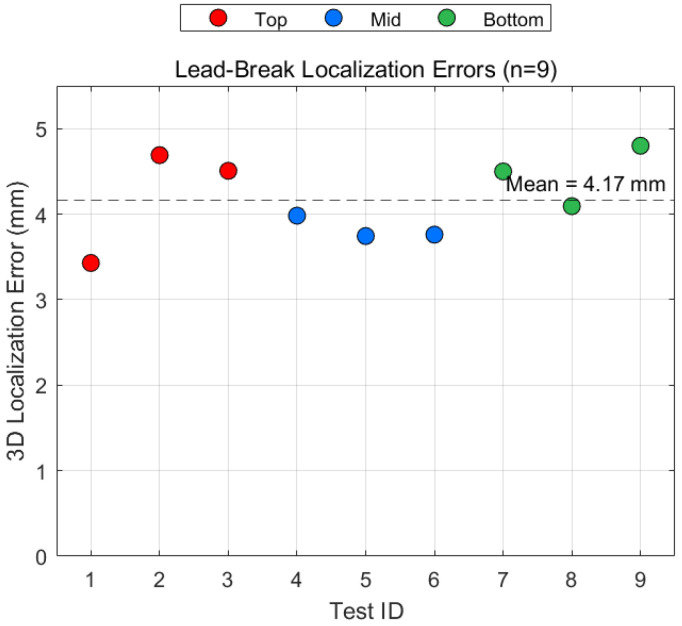
Determination of lead breakage positioning error.

As shown in Figure 14, the calculated source locations deviate from the true break points by 3.2–4.8 mm, with a mean absolute error of 4.1 ± 0.6 mm (≈2% of specimen height). This confirms sub-centimeter-level localization capability under the adopted acquisition and processing workflow.

### 4.2. Analysis of Sandstone Failure Characteristics and Location Results

**Figure 15:**
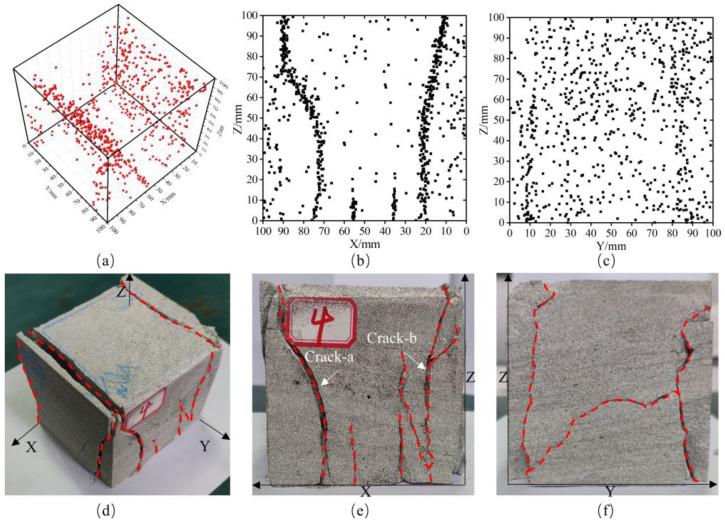
Source location results of loading and unloading failure of sandstone (**a**) 3D positioning space results; (**b**) XOZ surface positioning coordinate projection result; (**c**) YOZ surface positioning coordinate projection result; (**d**) Three-dimensional crack spatial morphology; (**e**) Spatial morphology of cracks on XOZ surface; (**f**) Spatial morphology of cracks on YOZ surface.

As shown in Figure 15, during the true triaxial loading-unloading failure experiment, in the triaxial loading phase, the inherent pores and fractures inside the sandstone are compacted and closed under external forces, resulting in a small number of scattered acoustic emission (AE) source location points within the specimen. After the triaxial stress reaches a constant load, the internal stress adjustment of the sandstone gradually slows down, and almost no new AE source location points are generated. In the loading-unloading failure phase, with the continuous loading of vertical stress, the sandstone undergoes initial fracture, forming two V-shaped fracture surfaces at both ends perpendicular to the direction of the minimum principal stress (σ_3_). The AE source location points induced by the fracture are significantly concentrated on these two fracture surfaces. As the vertical stress continues to increase, the sandstone experiences secondary fracture, further forming fracture surfaces perpendicular to the direction of σ_3_. Additionally, a small amount of fracture occurs at the corners due to stress concentration, and the induced AE source location points are clearly concentrated on the aforementioned fracture surfaces. The AE source location results of the loading-unloading failure demonstrate that under the conditions of vertical loading and lateral unloading, the sandstone undergoes severe damage only in the direction of the minimum principal stress (σ_3_).

**Figure 16:**
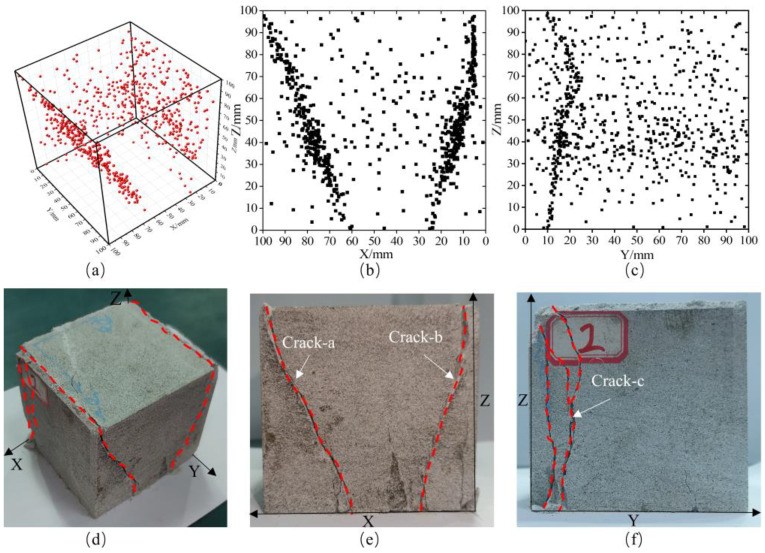
Source location results of loading failure of sandstone (**a**) 3D positioning space results; (**b**) XOZ surface positioning coordinate projection result; (**c**) YOZ surface positioning coordinate projection result; (**d**) Three-dimensional crack spatial morphology; (**e**) Spatial morphology of cracks on XOZ surface; (**f**) Spatial morphology of cracks on YOZ surface.

As shown in Figure 16, during the true triaxial loading failure experiment, in the triaxial loading phase, the inherent fine pores and fractures inside the sandstone are compacted and closed under external forces, resulting in a small number of relatively scattered acoustic emission (AE) source location points within the specimen. After the triaxial stress reaches a constant load, the internal stress adjustment of the sandstone gradually slows down, and almost no AE source location points induced by fracture are generated. In the loading failure phase, with continuous vertical loading and constant lateral confining pressure, the sandstone undergoes initial fracture as the stress continues to increase, forming two V-shaped fracture surfaces at both ends perpendicular to the direction of the minimum principal stress (σ_3_). The AE source location points induced by the fracture are significantly concentrated on these two fracture surfaces. Subsequently, the sandstone experiences secondary fracture, forming a V-shaped fracture surface at one end perpendicular to the direction of the intermediate principal stress (σ_2_), with the induced AE source location points concentrated on this fracture surface. The results demonstrate that under the conditions of vertical loading and constant lateral confining pressure, the sandstone first undergoes severe damage in the direction of the minimum principal stress (σ_3_), followed by minor damage in the direction of the intermediate principal stress (σ_2_).

It should be noted that the scale-dependent effects and discontinuity factors were not considered in this rock specimen-scale experimental study. The size effect is a core influencing factor in the analysis of fracture evolution laws. Due to scale limitations and the simplification of discontinuities such as bedding planes, small-scale specimens are difficult to reflect the real characteristics of rock mass fracture evolution under engineering-scale conditions. In future research, we will design and conduct larger-scale physical model experiments to closely approximate the rock mass scale in engineering sites, thereby mitigating the interference of the size effect on the analysis results. Meanwhile, discontinuities such as bedding planes will be introduced to accurately simulate their presence in natural rock masses, restore real rock mass conditions, and correct the analytical deviations caused by the combination of size effect and the absence of discontinuities. By optimizing the research direction through larger-scale experiments combined with discontinuity simulation, the limitations of the study caused by the size effect can be effectively addressed, making the analysis results of fracture evolution laws more valuable for engineering applications.

### 4.3. Characteristics of Spatial and Temporal Distribution in Each Stage of Positioning

**Figure 17:**
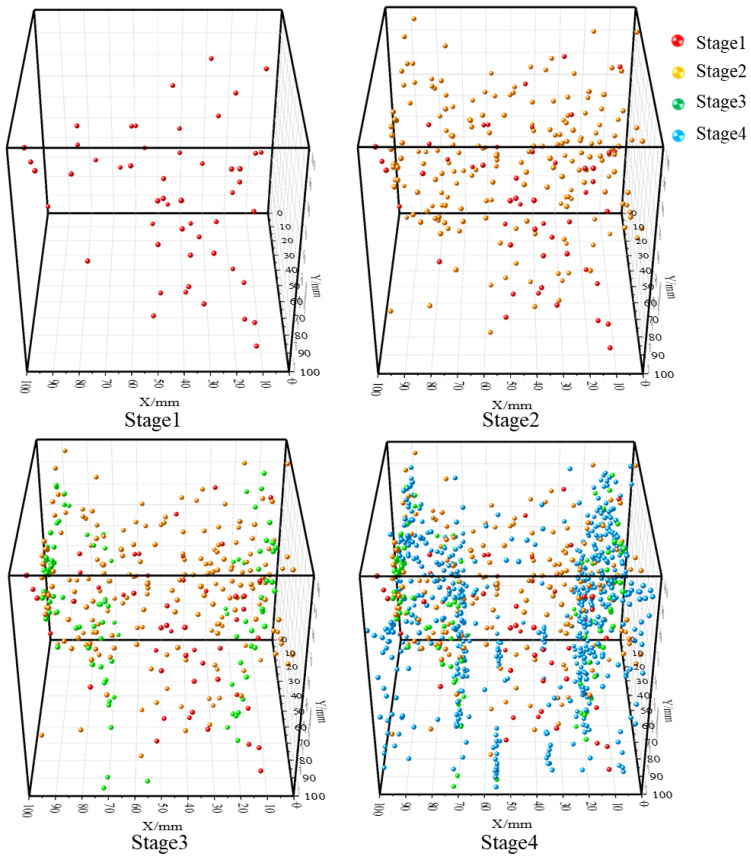
Source location results at different stages of unloading failure of sandstone.

As shown in Figure 17, during the true triaxial loading-unloading failure experiment of sandstone, in the first loading stage, a small number of AE (acoustic emission) source location points are generated inside the sandstone with a scattered distribution. Simultaneous triaxial loading compacts the pores and fractures within the sandstone. In the second stage, as the stress gradually increases, the sandstone continues to be compacted, and a small number of new AE source location points are generated. In the third stage, tensile stress is induced in the direction of the minimum principal stress (σ_3_) of the sandstone under the combined action of vertical loading and horizontal unloading. Cracks initiate and develop in the upper part of the sandstone, and the AE source location points concentrate at the cracks formed at both ends, indicating that cracks parallel to the vertical direction are formed on the upper and lower surfaces of the sandstone. In the fourth stage, with the continuous increase in vertical stress, the internal cracks of the sandstone start to extend from the upper and lower surfaces until they penetrate the sandstone to form a macroscopic fracture surface, and a large number of AE source location points also concentrate on this fracture surface.

**Figure 18:**
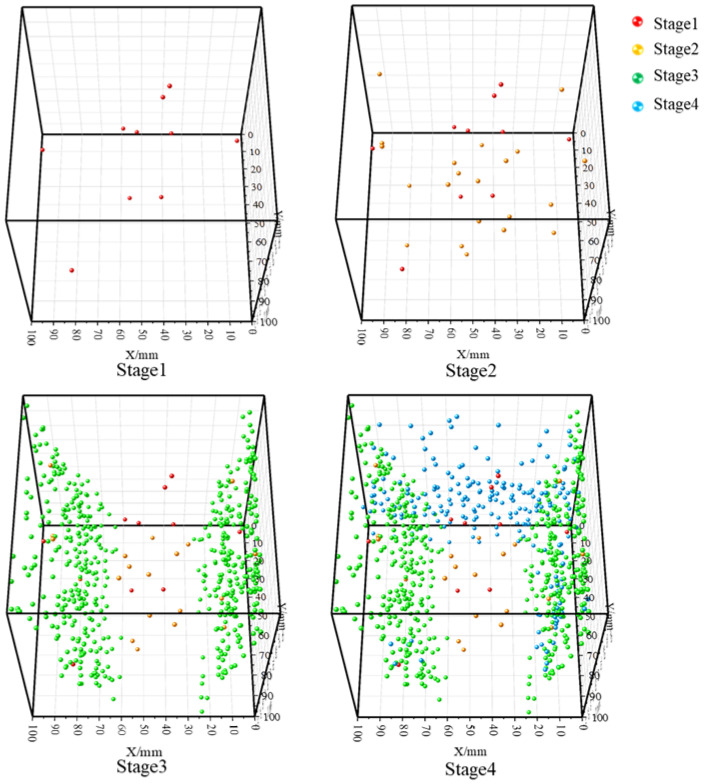
Source location results at different stages of loading failure of sandstone.

As shown in Figure 18, during the true triaxial loading failure experiment of sandstone, in the first stage, the sandstone is compacted under external forces, generating very few AE (acoustic emission) source location points that are scattered in distribution. In the second stage, entering the early elastic phase, almost no new AE source location points are generated inside the sandstone. In the early stage of the third stage, under the action of continuous vertical loading and constant lateral confining pressure, the sandstone undergoes initial fracture, forming two fracture surfaces at both ends perpendicular to the direction of the minimum principal stress (σ_3_). In the late stage of the third stage, as the vertical stress continues to increase, the sandstone experiences secondary fracture, forming an additional fracture surface at one end perpendicular to the direction of the intermediate principal stress (σ_2_), with AE source location points significantly concentrated on this fracture surface.

This indicates that under such stress conditions, for sandstone materials with good homogeneity, fracture surfaces with severe damage are first formed in the direction of the minimum principal stress (σ_3_), followed by minor damage in the direction of the intermediate principal stress (σ_2_), while almost no damage occurs in the direction of the maximum principal stress (σ_1_).

## 5. Conclusions

(1) Both the true triaxial loading and loading-unloading failure processes of sandstone exhibit four-stage evolutionary characteristics, with significantly differentiated temporal variation laws of acoustic emission (AE) signals corresponding to each stage. During the preloading stage, the rock mass is compacted, resulting in active AE signals with high amplitudes. In the formal loading stage, signals tend to be sparse and low-amplitude. In the failure stage, both the number and amplitude of signals increase significantly. Among these stages, the increase in cumulative AE energy and counts during the failure stage under the loading-unloading path is far greater than that in the preloading and formal loading stages, and the unloading process can induce rock mass fracture in advance and enhance the AE response. In contrast, under the pure loading path, the signal increase in the first two stages is minimal, while it rises significantly in the failure stage.
(2) The frequencies of AE waveforms during sandstone failure are concentrated in the range of 0–0.3 MHz, with dominant frequencies mainly distributed between 0 and 0.2 MHz. Amplitude peaks stably occur in the 0 MHz and 0.1 MHz frequency bands. High-amplitude waveforms correspond to the low-frequency range of 0–0.1 MHz, and their frequencies show a decreasing trend with increasing stress. Two low-frequency and high-amplitude regions are formed under the loading-unloading path, while only one such region appears under the pure loading path. There is no essential difference in the overall frequency distribution between the two paths.
(3) The loading path significantly affects the source mechanism and crack evolution mode of sandstone. Under the loading-unloading path, the loading of the maximum principal stress (σ_1_) and lateral unloading of the minimum principal stress (σ_3_) in Stage 3 promote the development of tensile failure cracks in the rock mass. Continuous loading in Stage 4 leads to crack propagation and coalescence, forming a macroscopic fracture surface. In contrast, under the pure loading path, vertical loading in Stage 3 induces the initiation of compression-shear cracks inside the rock mass. After continuous loading, the shear cracks coalesce and extend to the surface in Stage 4.
(4) The AE source location results have a good response relationship with the rock mass fracture process: during the preloading and formal loading stages, rock mass compaction only generates a small number of scattered location points; in the failure stage, a large number of concentrated location points appear, and these points are mostly clustered near fractures and fracture surfaces. This can effectively reflect the initiation, development process of cracks, and the propagation-coalescence characteristics of fracture surfaces.
(5) This study can provide important theoretical support and a scientific basis for microseismic monitoring and early warning of deformation and failure of surrounding rock in deep mine tunnels. For the tunnel excavation stage, the initial triaxial isostatic stress balance of the surrounding rock is disrupted, and it is transformed into a complex stress environment characterized by vertical loading, radial unloading, and constant strike stress through loading-unloading effects. For the post-excavation stage, the stabilized surrounding rock behind the working face is disturbed by subsequent mining activities, forming a complex stress path of vertical secondary loading. A three-dimensional analysis platform integrating time-frequency parameters, location density, and damage evolution is established to automatically identify characteristic patterns such as low-frequency high-amplitude discrete signals and high-frequency dense signals, and realize a graded alarm combined with early warning thresholds. This study provides a theoretical basis for the microseismic monitoring and early warning technology of surrounding rock deformation and failure under the above two scenarios.

## Figures and Tables

**Table 1:** Spatial layout and coordinates of AE sensors.

Sensor Number	Coordinate (mm)			Sensor Number	Coordinate (mm)			Acquisition Parameters Setting
	X	Y	Z		X	Y	Z	
N 1	30	0	30	N 6	70	100	30	Threshold:40 dB Sampling frequency: 1000 kHz Sampling time: 1024 μs
N 2	70	0	70	N 7	30	100	70	
N 3	100	30	30	N 8	20	100	20	
N 4	100	70	70	N 9	0	70	30	
N 5	100	80	20	N 10	0	30	70	

**Table 2:** Mechanical parameters of sandstone.

Rock Type	Uniaxial Compressive Strength (UCS)/MPa	Elastic Modulus/GPa	Tensile Strength /MPa	Poisson’s Ratio /μ	Cohesion C /MPa	Internal Friction Angle/°
Sandstone	22.687	3.099	2.751	0.157	8.063	39.83

## Data Availability

The data used to support the findings of this study are available from the corresponding author upon request.

## Abbreviations

The following abbreviations are used in this manuscript:

MS	Microseismic
AE	Acoustic emission
